# The acceptability and validity of self‐collected nasal swabs for detection of influenza virus infection among older adults in Thailand

**DOI:** 10.1111/irv.12471

**Published:** 2017-09-12

**Authors:** Sonal Goyal, Kriengkrai Prasert, Prabda Praphasiri, Malinee Chittaganpitch, Sunthareeya Waicharoen, Darunee Ditsungnoen, Siriluk Jaichuang, Kim A. Lindblade

**Affiliations:** ^1^ ASPPH/CDC Allan Rosenfield Global Health Fellow Atlanta GA USA; ^2^ Influenza Program Thailand Ministry of Public Health‐U.S. Centers for Disease Control and Prevention Collaboration Nonthaburi Thailand; ^3^ Nakhon Phanom Provincial Health Office Nakhon Phanom Thailand; ^4^ National Institute of Health Ministry of Public Health Nonthaburi Thailand; ^5^ Influenza Division U.S. Centers for Disease Control and Prevention Atlanta GA USA

**Keywords:** elderly, influenza, nasal swab, self‐collection, sensitivity, Thailand

## Abstract

**Background:**

Self‐collection of nasal swabs could improve the timeliness of influenza virus detection in older adults.

**Objectives:**

Measure the acceptability, adequacy, timeliness, and validity of self‐collected nasal swabs among adults >65 years in Thailand.

**Methods:**

Our evaluation consisted of two parts: a one‐month study among randomly selected, community‐dwelling older adults to simulate community‐based surveillance for acute respiratory infections (ARI); and a clinic study of older adults with ARI to evaluate the sensitivity and specificity of self‐collected nasal swabs for influenza virus infection compared with healthcare worker (HCW)‐collected nasal and nasopharyngeal swabs.

**Results:**

In the community study, 24% of participants experienced an ARI during the observation period. All (100%) participants with an ARI self‐collected nasal swabs within 72 hours of symptom onset of which 92% were considered adequate samples. In the clinic study, 45% of patients with ARI presented within 72 hours of symptom onset. The sensitivity of self‐collected nasal swabs for detection of influenza virus infection was 78% (95% CI 40‐97) compared to nasopharyngeal and 88% (95% CI 47‐100) compared to nasal swabs collected by HCWs. Specificity was 100% (95% CI 97‐100) compared to both methods. Self‐collection of nasal swabs was found acceptable by 99% of participants in both studies.

**Conclusions:**

Self‐collection of nasal swabs was acceptable to older adults in Thailand who were able to take adequate samples. Self‐collection of nasal swabs may improve the timeliness of sample collection but lower sensitivity will need to be considered.

## 1 INTRODUCTION

Influenza virus infection causes a range of illness from mild to severe disease and death, with the highest rates of morbidity and mortality among the very young and very old.^1^, ^2^, ^3^ Vaccination against influenza is the best way to prevent influenza‐related morbidity and mortality.^4^ Large population studies may be needed to evaluate vaccine effectiveness and to conduct influenza surveillance.^5^ However, the need to collect timely diagnostic samples during acute illness periods at a reasonable cost may be challenging in such studies. Self‐collection of nasal swabs may be one method to improve the detection of influenza cases in a timely and cost‐effective manner for epidemiologic studies and surveillance.

Self‐collection of nasal swabs involves provision of a swab kit to participants to be used at home to swab the anterior nares within 2‐3 days of developing an acute respiratory infection (ARI) when influenza virus is present at the highest concentration.^6^ After collection, swabs may be returned to study staff by mail, dropped off or collected at home by study staff. Several studies have shown self‐collection of nasal swabs among adults to be a feasible and reliable method for ARI surveillance.^5^, ^7^, ^8^, ^9^ The Flu Watch Cohort Study followed community cohorts in England over six successive influenza seasons and successfully obtained diagnostic nasal specimens through self‐collection of nasal swabs for more than 85% of ARI episodes.^5^ Additionally, a recent study among Swedish adults used nasal self‐swab samples mailed in by participants for virus detection and found that the influenza‐positive test results could be used to measure influenza seasonality.^7^ However, to our knowledge, there has not yet been a specific evaluation of self‐collection of nasal swabs by older adults, who may have reduced mobility and cognitive function that could limit their ability to collect valid nasal swabs.

Between 2005 and 2008 in Thailand, influenza virus infection resulted in an average of 7383 hospitalizations and 119 deaths annually among adults ≥50 years.^3^ During this same period, the annual incidence of influenza pneumonia in Thailand was highest in patients >75 years old. Although persons >65 years are eligible for free annual influenza vaccination in Thailand, national vaccine coverage in this age group is modest and only 20% of older adults in 2012 were vaccinated against influenza.^10^ Effectiveness of the influenza vaccine in preventing influenza‐related morbidity in older adults rarely has been measured in Thailand, and only once against laboratory‐confirmed influenza.^11^, ^12^ In preparation for a cohort study of the effectiveness of the inactivated influenza vaccine to prevent influenza‐associated morbidity among persons >65 years in Thailand, we conducted a pilot study to determine the acceptability, adequacy, timeliness, and validity of self‐collected nasal swabs.

## 2 METHODS

Our evaluation consisted of two parts: a community study to evaluate the acceptability, timeliness, and the adequacy of samples collected by community‐dwelling persons >65 years; and a clinic study to evaluate the acceptability of self‐swabbing, and the sensitivity and specificity of self‐collected nasal swabs for influenza virus detection compared to samples collected by healthcare workers (HCW).

### 2.1 Community study

The community study was conducted in one subdistrict of Nakhon Phanom Province. We took a systematic, random sample of persons >65 years using a list of all residents >65 years derived from a community census that was updated just prior to sampling. To ensure representativeness of the sample with respect to age, residents were ordered by age, first and last names and selected according to the sampling interval after a random start. Selected individuals were eligible for enrollment if they were non‐institutionalized, resident of the area for at least one year, able to communicate in Thai, and had access to a telephone. Participants were excluded if they were unable to communicate or understand instructions, paralyzed, or prone to nose bleeds.

At enrollment in February 2015, participants were provided with a nasal swab kit that included a foam‐tipped nasal swab (Puritan Medical Products Co., LLC, Guilford, ME, USA), a test tube with universal transport media ([UTM] Copan Diagnostics, Murrieta, CA, USA), and written instructions. Participants watched a video on self‐collection of nasal swabs (https://www.youtube.com/watch?v=QjzPWwXI1Ug) and received oral instruction from study staff on how to take and store a nasal swab. Each participant was instructed to collect and store a nasal swab as soon as possible after the onset of an ARI, defined as the onset of cough or worsening of chronic cough. Participants were instructed to insert the swab approximately one inch into their anterior naris for five‐seconds, turn and swirl the swab twice while touching the nasal cavity walls, slowly remove the swab, and place it tip‐first inside the test tube filled with UTM before securely closing the test tube. Participants were asked to keep the tube with UTM and nasal swab in their refrigerator before and after specimen collection due to high ambient temperatures. Participants were asked to call the study team to pick up the swab following an ARI. To remind participants to collect samples when they experienced an ARI, study staff contacted participants every week for the one‐month monitoring period. At the end of the monitoring period, participants who had not experienced an ARI were asked to collect a nasal swab on the last day of the study in order to evaluate the acceptability of the procedure and test for the presence of human cells.

### 2.2 Clinic study

The clinic study was conducted between February and December 2015 at four outpatient clinics in Nakhon Phanom Province among persons >65 years old seeking medical attention for an ARI, defined as having two or more of the following symptoms starting within the last seven days: cough or worsening of a chronic cough, measured (axillary temperature ≥38.5°C) or subjective fever, nasal congestion, and sore throat. The ARI case definition for the clinic was constructed to maximize the likelihood that the ARI was caused by influenza, whereas the ARI case definition for the community was designed to increase the chances of an ARI event to trigger self‐collection of a nasal swab. Individuals were eligible for enrollment if they were a resident of Nakhon Phanom and excluded if they were unable to communicate or understand instructions, prone to nose bleeds, or presented with severe illness that needed urgent medical attention. Patients who participated in the community study were not eligible for the clinic study.

Patients presenting to the outpatient department were screened for respiratory‐related symptoms and invited to enroll if they met eligibility criteria and the case definition. Participants watched the same video on self‐collection of nasal swabs and received oral and written instruction from project staff. Patients first swabbed their right anterior naris and then study HCWs collected a nasal swab from the left naris and a nasopharyngeal (NP) swab from either naris; swabs were stored in separate tubes and analyzed separately. Foam‐tipped swabs were used for all nasal samples, and flocked swabs (Copan Diagnostics, USA) were used for NP samples.

### 2.3 Outcomes acceptability, adequacy, feasibility, timeliness, and validity

Participants in both studies were interviewed by trained study staff after a nasal specimen was obtained to determine acceptability of self‐collection of nasal swabs based on self‐report. Participants in the clinic study additionally were asked to evaluate the acceptability of swabbing by HCWs. Questions were designed to determine participants’ ease and comfort level in collecting a nasal swab, their understanding of instructions, and general acceptability of self‐collection of nasal swabs. Participants rated each item on a three‐point scale as disagree, neutral, or agree.

The adequacy of self‐collected nasal swabs was evaluated among participants of the community study who reported an ARI during the one‐month observation period. An adequate sample was defined as: a nasal swab self‐collected within 72 hours of symptom onset, refrigerated after collection in a capped tube with UTM until retrieved by study staff, and testing positive for ribonuclease P (Rnase P), an indicator of the presence of human cells.^13^ A sample was positive for Rnase P if the cycle threshold (Ct) value from real‐time reverse transcriptase polymerase chain reaction (rRT‐PCR) test was <37.

The sensitivity and specificity of self‐collected nasal swabs for detection of influenza virus were measured in the clinic study among outpatients presenting with ARI.

### 2.4 Sample size

For the community study, we calculated a sample size of 150 persons >65 years old to measure the proportion who could produce an adequate nasal swab with a precision of +/− five percentage points, assuming a proportion of 90%, a Type I error rate of 5% and a combined refusal and dropout rate of 10%. For the clinic study, we calculated that a sample size of 19 ARI patients positive for influenza virus and 35 patients negative for influenza virus were needed, assuming a sensitivity of 95%, specificity of 90%, precision of +/− 10 percentage points, and a 5% Type I error rate.

### 2.5 Storage and laboratory analysis

In the community study, specimens were stored in participants’ own or a neighbor's refrigerator for less than 24 hours until they were collected by study staff with cool boxes at 2‐8°C. Both community and clinic samples were stored at 2‐8°C in a refrigerator at a centralized clinic, with storage temperatures monitored daily. In less than 24 hours, all the samples in the clinic refrigerator were placed into liquid nitrogen tanks. Samples were sent on liquid nitrogen weekly to the Thai National Institute of Health (NIH) for rRT‐PCR analysis. All specimens were tested by rRT‐PCR for the presence of Rnase P.^14^ Clinic specimens were also tested for influenza A and B viruses using standard protocols and Ct values from rRT‐PCR were recorded.^15^

### 2.6 Data analysis

Data were analyzed using sas version 9.3 (SAS Institute Inc., Cary, NC, USA). Only results from participants’ first episode of ARI in the community study were used to analyze acceptability and adequacy of self‐swabs. Paired *t* tests were used to compare Ct values between samples taken from the same patient. Chi‐square tests were used to compare proportions. Measurement of sensitivity and specificity compared the detection of influenza virus between self‐collected nasal swabs and two different gold standards: HCW‐collected nasal and HCW‐collected NP swabs. The exact binomial method was used to calculate 95% confidence intervals for sensitivity and specificity.

### 2.7 Human subjects

Written consent was obtained from all participants prior to enrollment. The study was approved by the Ethical Review Committee, Department of Disease Control, Ministry of Public Health (Nonthaburi, Thailand); the Centers for Disease Control and Prevention (Atlanta, GA, USA) relied on this committee for ethical approval.

## 3 RESULTS

### 3.1 Participant characteristics

Of the 150 participants selected for the community study, we enrolled 108 (72%; 12 could not be found, 26 met one or more exclusion criteria, and four declined to participate). Participants in the community study had a mean age of 73 years (range 65‐91; Table 1), 59 (55%) were female, 54 (50%) currently lived with a spouse, and 15 (14%) had received education past primary school. Participants in the community study experienced 26 (24%) cases of ARI within the 1 month period.

**Table 1:** Demographics of participants in the community and clinic studies

Characteristics	Community participants (n=108) N (%)
Age (y), mean (range)	73 (65‐91)
Female	59 (55%)
Marital status	
Single (never married)	6 (6%)
Currently married	54 (50%)
Divorced/separated	2 (2%)
Widowed	45 (42%)
Unknown	1 (1%)
Education	
None	0 (0%)
Attended or completed primary	92 (85%)
Attended or completed secondary	12 (11%)
Attended or completed post‐secondary	3 (3%)
Unknown	1 (1%)

	Clinic participants (n=127) N (%)
Age (y), mean (range)	73 (65‐95)
Symptoms	
Cough	124 (98%)
Fever	80 (63%)
Nasal congestion	85 (67%)
Sore throat	78 (61%)
Time since onset of symptoms	
<72 h	57 (45%)
3‐4 d	37 (29%)
5+ d	33 (26%)

There were 127 participants enrolled in the clinic study with a mean age of 73 years (range 65‐95; Table 1). Among the clinic participants, 124 (98%) experienced cough. Fever, nasal congestion, and sore throat were also common. Clinic visits and sample collection occurred <72 hours, 3‐4 days, and >5 days from symptom onset for 57 (45%), 37 (29%), and 33 (26%) of the participants, respectively.

### 3.2 Acceptability of self‐collected nasal samples

Among all participants in both studies, 232 (99%) found self‐collection of nasal swabs acceptable and 229 (97%) agreed that self‐collection of nasal swabs was easy to perform (Table 2). There were 38 (16%) participants who felt uncomfortable taking the swab themselves and 26 (11%) who found the instructions confusing. In the clinic study, the proportion of participants who felt uncomfortable when a HCW took either a nasal or NP swab (34, 27%) was higher than the proportion who felt uncomfortable when they self‐swabbed (22, 17%; *P*<.001).

**Table 2:** Acceptability of nasal swabbing as reported by participants

	Community (n=108) N (%)	Clinic (n=127) N (%)	Total (n=235) N (%)
Self‐collection of nasal swab			
Procedure was acceptable	108 (100)	124 (98)	232 (99)
Participant felt uncomfortable	16 (15)	22 (17)	38 (16)
Procedure was easy	107 (99)	122 (96)	229 (97)
Instructions were confusing	3 (3)	23 (18)	26 (11)
Participant remembered how to take the swab	106 (98)	‐	‐
Participant remembered how to store the swab	106 (98)	‐	‐
Collection by healthcare workers			
Procedure was acceptable	‐	120 (94)	‐
Participant felt uncomfortable with the nasal swab	‐	24 (19)	‐
Participant felt uncomfortable with the nasopharyngeal swab	‐	27 (21)	‐
Participant felt uncomfortable with either the nasal or the nasopharyngeal swab	‐	34 (27)	
I would prefer to take a nasal swab myself and not have it taken by study personnel	‐	50 (39)	‐

### 3.3 Adequacy of self‐collected nasal samples

Among the 26 ARI samples collected from community participants, 24 (92%) were considered adequate samples; 26 (100%) samples were collected within 72 hours of symptom onset, 26 (100%) met storage criteria, and 24 (92%) tested positive for Rnase P. There were 82 persons who took self‐collected nasal swabs at the end of the study, and 81 (99%) tested positive for Rnase P. In the clinic study, 127 (100%) samples had adequate levels of Rnase P, and the mean Ct values for Rnase P in self‐collected nasal swabs and HCW‐collected nasal swabs were similar (29.50 and 29.08, respectively; *P*=.06).

### 3.4 Sensitivity and specificity of nasal self‐swab samples to detect influenza virus

A total of 9 (15%) influenza virus infections were detected among 127 participants of the clinic study; 6 (10%) had influenza A (H3N2) and 3 (5%) had influenza B virus. The number of samples positive for influenza virus was 9 (100%) by HCW‐collected NP swabs, 8 (89%) by HCW‐collected nasal swabs and 7 (78%) by self‐collected nasal swabs. The sensitivity of self‐collected nasal swabs compared to HCW‐collected NP swabs was 78% (95% confidence interval [CI], 40‐97) and the specificity was 100% (95% CI, 97‐100; Table 3). The sensitivity of self‐collected nasal swabs compared to HCW nasal swabs was 88% (95% CI, 47‐100), and the specificity was 100% (95% CI, 97‐100).

**Table 3:** Comparison of the sensitivity and specificity of self‐collected nasal swabs to healthcare worker collected nasal and nasopharyngeal swabs among clinic outpatients for the detection of influenza A and B viruses by rRT‐PCR (n=127)

	Nasal self‐swab	
	n/N (Sensitivity [95% CI])	n/N (Specificity [95% CI])
HCW nasal swab	7/8 (88% [47‐100])	118/118 (100% [97‐100])
HCW nasopharyngeal swab	7/9 (78% [40‐97])	117/117 (100% [97‐100])

HCW, healthcare worker collected.

The Rnase P Ct values for the two self‐collected nasal swab samples discordant for influenza virus with the HCW‐collected NP swabs were higher (indicating a lower quantity of human cells) than for the NP samples (Table 4). Similarly, the sample that was discordant for influenza virus between HCW‐collected NP and HCW‐collected nasal swab had a higher Rnase P Ct value in the sample negative for influenza virus. The two discordant samples were both collected on the third day after symptom onset.

**Table 4:** Comparison of ribonuclease P cycle threshold (Rnase P Ct) values for two samples discordant for influenza virus between sample types

	Participant 1 Rnase P Ct (influenza virus +/−)	Participant 2 Rnase P Ct (influenza virus +/−)
HCW NP swab	23.83 (+)	27.67 (+)
HCW nasal swab	20.99 (+)	30.43 (−)
Nasal self‐swab	26.29 (−)	29.04 (−)

HCW, healthcare worker collected; NP, nasopharyngeal.

## 4 DISCUSSION

We found self‐collection of nasal swabs to be highly acceptable to adults >65 years in Thailand and most (92%) were able to take and store an adequate sample. Timeliness of sample collection relative to symptom onset was better with self‐collection of nasal swabs than with samples collected from persons with ARI seeking medical attention. In the community study, which we believe represents results that could be expected from research or surveillance studies using self‐collection of nasal swabs, 100% of nasal swabs were self‐collected within 72 hours of symptom onset, compared to results from the clinic study, which is more typical of facility‐based surveillance, in which only 45% of patients presented within 72 hours of symptom onset.

Two previous studies that investigated the acceptability of nasal self‐collection of nasal swabs for detection of viral pathogens among adults between 18 and 69 years of age in Germany showed similar rates of acceptability, ease of self‐collection of nasal swabs, and comfort level as we found in this evaluation.^8^, ^16^ In Smieja et al.^17^, asymptomatic adults were asked to take two self‐collected nasal swabs and the authors found that 87% and 98% of first and second self‐collected nasal swabs, respectively, had adequate cell counts (defined as >25 cells/high powered field). Akmatov et al.^16^ found that the mean β‐actin DNA concentration (a proxy for the presence of human epithelial cells) was higher among self‐collected nasal swabs than healthcare worker‐collected swabs, indicating that self‐collected swabs were adequate for detection of influenza.

When compared with both HCW‐collected nasal and NP swabs in this study, however, self‐collected nasal swabs were less sensitive for detecting influenza viruses. Sensitivity may have been reduced by time since symptom onset as 26% of clinic samples were collected five or more days after symptom onset when influenza virus is no longer at its highest concentration and as detectable by rRT‐PCR.^6^ While nasopharyngeal aspirates or swabs have been considered the best samples for influenza detection in epidemiologic studies, a paired comparison of HCW‐collected nasal and NP swabs for influenza virus detection by PCR in the United States found no significant difference between the two methods^18^ although the sensitivity of nasal swabs was lower. Prior studies comparing nasal self‐swabs to HCW‐collected nasal swabs among adults have shown high sensitivity and specificity of nasal self‐swabs.^16^, ^19^ Dhiman et al. found a 95% qualitative agreement, a measurement that shows how close observed outcomes are to expected outcomes while controlling for chance, between self‐collected nasal swabs and HCW‐collected nasal swabs among adults reporting to the emergency department with influenza‐like illness.^19^ Akmatov et al. found a combined 100% sensitivity and 98% specificity for 15 respiratory viral pathogens between self‐collected nasal swabs and HCW‐collected nasal swabs among adults experiencing ARI.^16^ One study compared flocked mid‐turbinate self‐collected nasal swabs to HCW‐collected NP swabs among adult participants experiencing ARI in Canada and found that self‐collected nasal swabs had 90% combined sensitivity for detecting five respiratory viruses, which included three influenza virus positives in 29 total positive cases.^20^

In our study, the findings from two samples discordant for influenza virus detection between self‐collected nasal swabs and HCW‐collected nasal and NP swabs, despite adequate amounts of Rnase P, suggest that participants may not have been swabbing far enough up the nasal cavity. Additional support and explanations by study staff could help to rectify this deficiency. The difference in swab type (Puritan foam‐tipped swabs for nasal swabbing versus Copan flocked swabs for NP swabbing) may also account for the difference in findings between sample collection methods.

A strength of this study was measurement of sample adequacy using a community‐based study design that reflects similar conditions to what would be present in a community‐based surveillance or research study. As a result of low influenza transmission during our study, we did not have adequate numbers of influenza virus infections to measure sensitivity and specificity precisely or to stratify results by influenza type/subtype. Our study was not able to enroll all eligible individuals who presented to the clinic with ARI due to resource limitations, although we do not believe convenience sampling introduced a systematic enrollment bias.

Although self‐collected nasal swabs detected fewer influenza virus infections than HCW‐collected samples, we believe that the increased ease and acceptability of self‐collected nasal swabs will overcome sensitivity limitations to make self‐collection of nasal swabs a preferred method for influenza detection in community studies of older adults in Thailand. Reduced diagnostic sensitivity should be incorporated into sample size calculations to insure adequate power. Acceptability and feasibility of using self‐collected nasal swabs in other populations should be evaluated. In conclusion, self‐collection of nasal swabs could improve the timeliness of sample collection in population‐based surveillance and research for influenza among older adults in Thailand.

## CONFLICT OF INTEREST

There was no conflict of interest.

## DISCLAIMER

The opinions expressed by authors contributing to this journal do not necessarily reflect the opinions of the Centers for Disease Control and Prevention or the institutions with which the authors are affiliated.
